# Updated protocol: The effects of problem‐oriented policing on crime and disorder: An updated systematic review

**DOI:** 10.1002/cl2.1005

**Published:** 2019-07-25

**Authors:** David Weisburd, Joshua C. Hinkle, Cody Telep

**Affiliations:** ^1^ Hebrew University Jerusalem Israel; ^2^ George Mason University Fairfax Viginia; ^3^ Department of Criminology and Criminal Justice Georgia State University Atlanta Georgia; ^4^ School of Criminology and Criminal Justice Phoenix Arizona

## 1 BACKGROUND

### 1.1 The problem, condition, or issue

Problem‐oriented policing has garnered a great deal of attention since it was first proposed by Herman Goldstein in 1979. The core of the model is a shift from police operating in a reactive, incident‐driven way (primarily responding to calls for service) to a model that requires the police to be proactive in identifying underlying problems that can be targeted to alleviate crime and disorder at their roots. Problem‐oriented policing can be thought of as a process rather than a specific intervention. As such, problem‐oriented policing can work independently or simultaneously with other modern policing innovations (hot spots policing, focused deterrence etc.) to address problems of crime and disorder. While the ability of problem‐oriented policing to target an array of different issues makes it widely applicable, the plethora of different interventions that may qualify as problem‐oriented policing make generalizing research on its effect difficult. The current study will provide an updated systematic review of the effectiveness of problem‐oriented policing in reducing crime and disorder. An earlier Campbell review by three of the same authors covered studies published through 2006 (Weisburd, Telep, Hinkle, & Eck, 2008; 2010); this updated review will add studies published from 2006 to 2018.

### 1.2 The intervention

Since its initial proposition, the problem‐oriented policing model has been further articulated by Eck and Spelman (1987) whose work in Newport News produced the SARA model. SARA is an acronym representing four steps they suggest police should follow when implementing problem‐oriented policing. “Scanning” is the first step, and involves the police identifying and prioritizing potential problems in their jurisdiction that may be causing crime and disorder. After potential problems have been identified, the next step is “Analysis.” This involves the police analyzing the identified problem(s) so that appropriate responses can be developed. The third step, “Response,” has the police developing and implementing interventions designed to solve the problem(s). Finally, once the response has been administered, the final step is “Assessment” which involves assessing the impact of the response on the targeted problem(s).

For example, a police agency may determine that drug‐related crime is on the rise in their jurisdiction, constituting a problem in need of prioritization (Scanning phase). Further examination of the nature of drug‐related crime may reveal problem areas and times (Analysis phase). Based on this analysis, the agency may choose to direct increased patrol and enforcement to the specific areas deemed problematic, and at the specific times deemed problematic (Response phase). After a period of time the agency may compare drug related crime in the jurisdiction as a whole, as well as in the targeted areas, from before and after the response was implemented (Assessment phase). This process in general, rather than the specific problem or response chosen, represents the core concept of problem‐oriented policing. Thus, a diverse set of variations in problems, responses, and length of interventions are possible.

### 1.3 How the intervention might work

A 2004 report from the National Research Council offered the following description of problem‐oriented policing and how the SARA model works in practice.

The heart of problem‐oriented policing is that this concept calls on police to analyze problems, which can include learning more about victims as well as offenders, and to consider carefully why they came together where they did. The interconnectedness of person, place, and seemingly unrelated events needs to be examined and documented. Then police are to craft response that may go beyond traditional police practices…Finally, problem‐oriented policing calls for police to assess how well they are doing. Did it work? *What* worked, exactly? Did the project fail because they had the wrong idea, or did they have a good idea but fail to implement it properly? (Committee to Review Research on Police Policy and Practice, 2004: 91)

It is hypothesized that problem‐oriented policing affects change in problem outcomes through an increased knowledge of, and responsiveness to, the specificity with which a particular problem operates. Using this process as a policing framework should lead agencies to think and act in ways that go beyond their normal day‐to‐day operations. Furthermore, the assessment of results should lead to refinement and improvement in subsequent efforts.

### 1.4 Why it is important to do the review

Prior to the original Campbell review of problem‐oriented policing (Weisburd et al., 2008; 2010), earlier narrative reviews had concluded that research was consistently supportive of the capability of problem solving to reduce crime and disorder (e.g., Committee to Review Research on Police Policy and Practice, 2004; Weisburd & Eck, 2004). These conclusions were drawn largely from a number of quasi‐experiments going back to the mid‐1980s that consistently demonstrated that problem solving can reduce fear of crime (Cordner, 1986), violent and property crime (Eck & Spelman, 1987), firearm‐related youth homicide (Kennedy, Braga, Piehl, & Waring, 2001) and various forms of disorder, including prostitution and drug dealing (Capowich & Roehl, 1994; Eck & Spelman, 1987; Hope, 1994). For example, a quasi‐experiment in Jersey City, New Jersey, public housing complexes (Green‐Mazerolle, Ready, Terrill, & Waring, 2000) found that police problem‐solving activities caused measurable declines in reported violent and property crime, although the results varied across the six housing complexes studied. In another example, Clarke and Goldstein (2002) report a reduction in thefts of appliances from new home construction sites following careful analysis of this problem by the Charlotte‐Mecklenburg Police Department and the implementation of changes in building practices by construction firms.

In addition to the quasi‐experimental work that preceded the earlier systematic review, two experimental evaluations of applications of problem solving in hot spots suggested its effectiveness in reducing crime and disorder.^1^ In a randomized trial with Jersey City violent crime hot spots, Braga et al. (1999) reported reductions in property and violent crime in the treatment locations. While this study tested problem‐solving approaches, it is important to note that focused police attention was brought only to the experimental locations. Accordingly, it is difficult to distinguish between the effects of bringing focused attention to hot spots and that of such focused efforts being developed using a problem‐oriented approach. The Jersey City Drug Market Analysis Experiment (Weisburd & Green, 1995) provided more direct support for the added benefit of the application of problem‐solving approaches in hot spots policing. In that study, a similar number of narcotics detectives were assigned to treatment and control hot spots. Weisburd and Green (1995) compared the effectiveness of unsystematic, arrest‐oriented enforcement based on ad hoc target selection (the control group) with a treatment strategy involving analysis of assigned drug hot spots, followed by site‐specific enforcement and collaboration with landlords and local government regulatory agencies, and concluding with monitoring and maintenance for up to a week following the intervention. Compared with the control drug hot spots, the treatment drug hot spots fared better with regard to disorder and disorder‐related crimes.

Evidence of the effectiveness of situational and opportunity‐blocking strategies, while not necessarily police based, provides indirect support for the effectiveness of problem solving in reducing crime and disorder. Problem‐oriented policing has been linked to routine activity theory, rational choice perspectives, and situational crime prevention (Clarke, 1992a, 1992b; Eck & Spelman, 1987). Past reviews of prevention programs designed to block crime and disorder opportunities in small places found that most of the studies report reductions in target crime and disorder events (Eck, 2002; Poyner, 1981; Weisburd, 1997). Furthermore, many of these efforts were the result of police problem‐solving strategies. We note that many of the studies reviewed employed relatively weak designs (Clarke, 1997; Eck, 2002; Weisburd, 1997).

Building off this work, the earlier Campbell systematic review of the effectiveness of problem‐oriented policing (Weisburd et al., 2008; 2010) identified a total of 10 studies that met the Campbell criterion for inclusion—four randomized experiments and six quasi‐experiments. Overall, the findings of this review largely reinforced those of the earlier narrative reviews and more general assumptions of the effectiveness of problem‐oriented policing. Specifically, the authors noted “[w]hether we used a more conservative mean effect size approach or examined the largest effects on crime and disorder reported, we found that POP approaches have a statistically significant effect on the outcomes examined. Importantly, the results are similar whether we look at experimental or nonexperimental studies” (Weisburd et al., 2010; p. 162). However, they also noted that effect sizes were relatively modest, ranging between .10 and .20 (small effect sizes by Cohen's standards). The authors also pointed to a host of other factors that should be examined in future reviews after more evaluations of POP had been conducted, including examining the impact of overlapping police interventions. For instance, many POP studies involve elements of hot spots policing and vice versa, but having only 10 eligible studies precluded the original review from examining those types of issues. We note that a recent review of the National Research Council concluded, drawing heavily from our previous review, that problem‐oriented policing was effective (Weisburd & Majmundar, 2018).

The findings of this updated review may help to shed further light on the ability of POP to reduce crime and disorder problems by analysing an increased base of empirical research on POP interventions. As empirical knowledge on POP's effectiveness increases police agencies may be able to better to determine ways to identify and respond to the various problems occurring in their jurisdictions.

## 2 OBJECTIVES

The objective of this updated systematic review is to synthesize the extant empirical evidence (published and unpublished) on the effects of problem‐oriented policing on crime and disorder, including all such works produced after the searches were conducted for the original review (Weisburd et al., 2008; 2010). The review aims to answer the following questions: Is problem‐oriented policing effective in reducing crime and disorder?; Does problem‐oriented policing have differential impacts across different types of crime and disorder?; Do specific types of problem‐oriented policing approaches have different effects on crime and disorder? As such, the primary question of this review is concerned with crime and disorder outcomes of problem‐oriented policing. Nonetheless, when data are available we will collect information on the cost effectiveness of problem‐oriented policing programs or other secondary outcomes such as its impacts on police legitimacy and fear of crime.

## 3 METHODOLOGY

### 3.1 Criteria for including and excluding studies

The scope of this review is experimental and quasi‐experimental studies of problem‐oriented policing. The preliminary eligibility criteria are as follows:

1. The study must be an evaluation of a problem‐oriented policing intervention. For this it is necessary to develop an operational definition of problem‐oriented policing. For this review, only police interventions following the basic tenets of the SARA model outlined above will be eligible for inclusion. This is to say that such interventions must involve the identification of a problem believed to be related to crime and/or disorder outcomes, the development and administration of a response specifically tailored to this problem and an assessment of the effects of the response on a crime or disorder outcome.
2. Eligible studies must meet the methodological criterion used for inclusion in the Global Policing Database (GPD; www.gpd.uq.edu.au). This will be discussed in detail in Section 3.3 below.
3. The study must report on the impacts of POP on at least one crime/disorder outcome.

### 3.2 Search strategy

The search for this updated review will be led by the Global Policing Database (GPD) research team at the University of Queensland (Elizabeth Eggins and Lorraine Mazerolle) and Queensland University of Technology (Angela Higginson). The University of Queensland is home to the GPD (see http://www.gpd.uq.edu.au), which will serve as the main search location for this review. The GPD is a web‐based and searchable database designed to capture all published and unpublished experimental and quasi‐experimental evaluations of policing interventions conducted since 1950. There are no restrictions on the type of policing technique, type of outcome measure or language of the research (Higginson, Eggins, Mazerolle, & Stanko, 2015). The GPD is compiled using systematic search and screening techniques, which are reported in Higginson et al. (2015) and summarized in Appendices A and B. Broadly, the GPD search protocol includes an extensive range of search locations to ensure that both published and unpublished research is captured across criminology and allied disciplines.

To capture studies, we will use problem‐oriented policing terms to search the GPD corpus of full‐text documents that have been screened as reporting on a quantitative impact evaluation of a policing intervention. Specifically, we will use the following terms to search the title and abstract fields of the corpus of documents published from January 2006 through December 2018:

1. "problem‐orient*”
2. "problem orient*"
3. “problem solv*”
4. SARA
5. scan*
6. "problem focus*"
7. “problem ident*”
8. “ident* problem*”
9. “situational crime prevent*”
10. POP

Several additional strategies will be used to extend the GPD search. First, we will perform forward citation searches for works that have cited seminal problem oriented policing studies.^2^ Second, we will perform hand searches of 2017 and 2018 volumes of leading journals in the field to identify any recent studies that may not yet be indexed in the GPD and other databases.^3^ Third, we will review the Center for Problem‐Oriented Policing website for all Tilley Award and Goldstein Award winners and submissions.^4^ Fourth, after finishing the above searches and reviewing the studies as described later, we will e‐mail the list to leading policing scholars knowledgeable in the area of problem‐oriented policing (see list in Appendix D). This is likely to identify studies the above searches missed, as these experts may be able to refer us to eligible studies missing from our list, particularly unpublished pieces such as dissertations and smaller research reports.

Several strategies will be used to obtain full‐text versions of the studies found through searches of the various abstract databases listed above. First, we will attempt to obtain full‐text versions from the electronic journals available through the George Mason University, Georgia State University and Arizona State University libraries. When electronic versions are not available, we will use print versions of journals available at the library. If the journals are not available, we will make use of the Interlibrary Loan Office (ILL) to try to obtain the journal from the libraries of other area schools. If these methods do not work, we will contact the author(s) of the article and/or the agency that conducted and/or funded the research to try to get a copy of the full‐text version of the study.

### 3.3 Description of methods used in primary research

The studies included in this review will use methodologies that are variations of a treatment versus comparison group research designs with a post‐test measure. Some studies may have additional follow‐up comparisons. Specifically, this review will include randomized experimental studies and a variety of types of quasi‐experimental studies using the criterion specified for inclusion of studies into the GPD. These criteria will also be used for screening for eligibility of studies found through our hand searches of journals and our searches of the publications from the agencies listed above. The criterion in the GPD protocol specify the following types of research designs as eligible for inclusions (Mazerolle, Eggins, & Higginson, 2016; p. 47–48):

1. Randomized experimental designs (RCTs)
2. The following “strong” quasi‐experimental designs:
  a. Regression discontinuity designs
  b. Matched control group designs with or without pre‐intervention baseline measures (propensity or statistically matched)
  c. Unmatched control group designs with pre‐intervention measures (difference‐in‐difference analysis)
  d. Short interrupted time‐series designs with control group (less than 25 preintervention and 25 postintervention observations (Glass, 1997)
  e. Long interrupted time‐series designs with or without control group ≥ 25 preintervention and postintervention observations (Glass, 1997)

1. The following “weak” quasi‐experimental designs:
  a. Unmatched control group designs with pre‐post intervention measures which allow for difference‐in‐difference analyses
  b. Unmatched control group designs without preintervention measures where the control group has face validity
  c. Raw unadjusted correlational designs where the variation in the level of the intervention is compared to the variation in the level of the outcome
  d. Treatment‐Treatment Designs

This review is excluding single group designs with preintervention and postintervention measures. These designs are highly subject to bias and threats to internal validity. As the earlier review of POP indicated, this is especially true in this area. Pre‐post studies were collected during that study for a separate analysis and results showed that while these studies had mostly positive and sizeable impacts on crime, they were highly subject to bias toward positive outcomes because a number of them were submissions for consideration for the Goldstein and Tilley awards for excellence in problem‐oriented policing. This, combined with inherent difficulty in publishing null findings from less rigorous research designs makes it difficult to draw strong conclusions from the body of pre‐post evaluations of POP. Such work is important as POP often involves addressing single problems. However, reviewing these studies in a Campbell Systematic review is not appropriate given the emphasis on only including rigorous experimental and quasi‐experimental designs. We know from the past review that there is promising case study evidence supporting the use of POP to address individual problems. The purpose of this review is to see whether strong methodological approaches continue to yield positive crime prevention outcomes for POP. The inclusion of case studies would not add to this goal.

The unit of analysis will be “problems” variously identified. In general, the outcomes will be drawn from data in geographic areas of varying size (possibly ranging from as small as street segments/blocks to as large as whole police districts or even cities) or groups of people targeted by the intervention or serving as a control group. The studies will also vary in their method of assignment to treatment and comparison areas. A small number may use randomized methods to assign areas to groups. Quasi‐experiments in which a handful of areas are assigned to treatment or control by a police department and/or research team (or where comparison areas are chosen after the fact) will be more common. Additionally, studies may use pre‐post designs in which there are no geographical comparison areas or control groups of individuals, as the preintervention time period in the target site is used as the comparison. As above, these simple pre‐post studies will not be included in this updated review.

All eligible studies will include a post‐intervention measure of crime and disorder. These can include measures of overall crime/disorder, or measures of individual categories of crime/disorder (i.e. homicide or robbery). These measures will largely be obtained from official police data such as calls for service, arrests and/or crime incident reports. However, it is possible that some studies may use alternative measures such as researcher observations of crime/disorder or self‐report measures of crime/delinquency. We do not expect to find many studies that allow for a cost benefit analysis, though this will not be known until the review is conducted. Other outcome measures such as fear of crime, citizen attitudes toward police etc. may be measured in the studies, though they are not generally primary outcomes of problem‐oriented policing.

### 3.4 Criteria for determination of independent findings

Many studies are expected to report multiple outcomes. An important problem in that case is how to treat such outcomes so that analyses will not include dependent outcomes in the same analysis. For example, some studies may report on multiple crime/disorder outcomes in the same target site. For cases such as this with multiple findings from the same sample, each will be examined independently to decide how to either combine the findings or to choose the one that best represents the study.

While it is likely that most interventions will be designed to deal with a specific problem, some may also target some secondary problems and report outcomes for these as well. In these cases the effect size for the primary problem will be reported.

In the case of a single study with multiple sites within the same jurisdiction, and reliant on the same police program, the results will be treated as multiple outcomes in the same study and will be averaged across sites. An example of this would be a study reporting on the effects of problem‐oriented policing on crime in multiple target sites in one city. Such cases will be treated as one study with sub units.

In the case where one POP program is designed to deal with multiple problems and reports outcomes for each of these problems, we will code all primary outcomes identified by study authors, and will report findings using the maximum, average, and minimum effect sizes to offer context for assessing the range of effect sizes for such studies. The same strategy will be used for any studies reporting the same outcome multiple times with different types of data (i.e. a study evaluating the impact of a POP program on robbery may report the outcome measured by robbery incidents, arrests and calls for service).

### 3.5 Details of study coding categories

As the GPD search process outlined above already screens the literature for policing evaluations with experimental and quasi‐experimental designs, our screening of full text results is limited to screening for studies of POP (defined as roughly following the tenets of the SARA model) that report impacts on at least one crime or disorder outcome. Our screening of studies identified through our gray literature searches will have to first screen for studies that used the experimental or quasi‐experimental methods listed above, in addition to verifying that they meet our definition of POP and report on a relevant outcome.

All eligible studies will be coded (see coding protocol attached in Appendix C) on a variety of criteria (including details related to them) including:

1. Reference information (title, authors, publication etc.)
2. Nature of description of selection of site, problems etc.
3. Nature and description of selection of comparison group or period
4. The unit of analysis
5. The sample size
6. Methodological type (randomized experiment or quasi‐experiment)
7. A description of the POP intervention
8. Dosage intensity and type
9. Implementation difficulties
10. The statistical test(s) used
11. Reports of statistical significance (if any)
12. Effect size/power (if any)
13. The conclusions drawn by the authors

Two members of the research team will independently code each eligible study. Where there are discrepancies, one of the lead authors (Dr. Weisburd, Hinkle or Telep) will review the study, discuss the coding decisions with the original coders and determine the final coding decision.

### 3.6 Statistical procedures and conventions

Meta‐analytic procedures will be used to combine data from studies. For eligible studies, with enough data present, effect sizes will be calculated using the standardized measures of effect sizes as suggested in the meta‐analytic literature (e.g., see Lipsey & Wilson, 2001). Mean effect sizes will be computed across studies and we will use a correction such as the inverse variance weight for computing the associated standard error. Though we will examine the Q statistic to assess heterogeneity of effect sizes across studies, it is our initial assumption that effect size is a random factor in our analysis. As such, we will implement a random effects model for all analyses involving effect sizes. This is the case because POP strategies are diverse, and they are brought to ameliorate different types of problems. The common factor is the process used by the police. In this context we believe that a mixed effects model will be most appropriate in analyzing the effectiveness of POP outcomes.

We also hope to examine contextual or moderating features of POP. Though it is difficult to know at the outset, we think it important to assess the differential effects of POP across different types of problems and across different types of treatments. We are also interested in whether the strength of the effect varies across departments or other contextual variables. If we identify enough relevant studies for statistical analysis, to assess this we will use the analog to the ANOVA method of moderator analysis (see Lipsey & Wilson, 2001) for categorical moderator variables and meta‐analytic regression analysis for continuous moderator variables or analyses involving multiple moderators.

Finally, publication bias is a concern in every meta‐analysis. As such, we will use traditional methods to test for the sensitivity of the findings to publication bias in the experimental and quasi‐experimental studies. These methods will include a comparison of the mean effect size for published and unpublished studies and a trim‐and‐fill analysis.

### 3.7 Treatment of qualitative research

We do not plan to include qualitative research.

### 3.8 Roles and responsibilities

1. Content: Weisburd, Hinkle, Telep
2. Systematic review methods: Weisburd, Hinkle, Telep
3. Statistical analysis: Hinkle, Telep
4. Information retrieval: GPD Team (Elizabeth Eggins, Lorraine Mazerolle, Angela Higginson), Hinkle, graduate research assistant(s)

## SOURCES OF SUPPORT

### External funding

Support for this review has been provided from The Police and Crime Commissioner via a subaward agreement between the Campbell Collaboration and George Mason University.

## DECLARATIONS OF INTEREST

Professor Weisburd has been an evaluator of problem‐oriented policing programs, including the Jersey City Drug Market Analysis Experiment and was lead author on the earlier Campbell review of POP (Weisburd et al., 2008; 2010). He has also published a review of police effectiveness in the ANNALS (Weisburd & Eck, 2004) which was based on his work at the National Research Council. The narrative review suggested that POP programs do have a positive crime and disorder outcome. That review provided the basis for Professor Weisburd's interest in carrying out the original systematic review. More recently Professor Weisburd chaired the National Research Council review of proactive policing, which concluded that POP was effective (based in good part on the Campbell review). Professor Weisburd would not be uncomfortable if the findings showed that the narrative review was incorrect or that findings have changed since the original systematic review.

Professors Hinkle and Telep were also authors on the original review of POP. Dr. Telep is also an author of a forthcoming guide on hot spots policing for the Center for Problem‐Oriented Policing (Telep & Hibdon, 2019). Like Professor Weisburd, they would not be uncomfortable if the updated review results in findings that differ from the original systematic review.

## PRELIMINARY TIMEFRAME

The review process will adhere to the following schedule:

Search for published and unpublished studies Spring 2019

Relevance assessments Spring to Early Summer 2019

Coding of eligible studies Summer 2019

Statistical analysis Summer to Fall 2019

Presentation of preliminary findings at ASC Fall 2019

Preparation of report Winter to Spring 2020

Submission of completed report Spring‐Early Summer 2020

## PLANS FOR UPDATING THE REVIEW

The authors expect to update the review every five to ten years depending on a sense of trends for experimental and quasi‐experimental evaluations of POP being funded and conducted. Many of these are highly visible as they are often published in top journals in the field.

## AUTHOR DECLARATION

### Authors’ responsibilities

By completing this form, you accept responsibility for preparing, maintaining and updating the review in accordance with Campbell Collaboration policy. Campbell will provide as much support as possible to assist with the preparation of the review.

A draft review must be submitted to the relevant Coordinating Group within two years of protocol publication. If drafts are not submitted before the agreed deadlines, or if we are unable to contact you for an extended period, the relevant Coordinating Group has the right to de‐register the title or transfer the title to alternative authors. The Coordinating Group also has the right to de‐register or transfer the title if it does not meet the standards of the Coordinating Group and/or Campbell.

You accept responsibility for maintaining the review in light of new evidence, comments and criticisms, and other developments, and updating the review at least once every five years, or, if requested, transferring responsibility for maintaining the review to others as agreed with the Coordinating Group.

### Publication in the Campbell Library

The support of the Coordinating Group in preparing your review is conditional upon your agreement to publish the protocol, finished review, and subsequent updates in the Campbell Library. Campbell places no restrictions on publication of the findings of a Campbell systematic review in a more abbreviated form as a journal article either before or after the publication of the monograph version in Campbell Systematic Reviews. Some journals, however, have restrictions that preclude publication of findings that have been, or will be, reported elsewhere and authors considering publication in such a journal should be aware of possible conflict with publication of the monograph version in Campbell Systematic Reviews. Publication in a journal after publication or in press status in Campbell Systematic Reviews should acknowledge the Campbell version and include a citation to it. Note that systematic reviews published in Campbell Systematic Reviews and co‐registered with Cochrane may have additional requirements or restrictions for co‐publication. Review authors accept responsibility for meeting any co‐publication requirements.

**I understand the commitment required to undertake a Campbell review, and agree to publish in the Campbell Library. Signed on behalf of the authors**:

**Table:** 

**Form completed by:** David Weisburd	**Date:** 19 February 2018

## Supporting information

Supplementary information

## Footnotes

1 A systematic review of “hot spots policing” has been conducted by Anthony Braga and colleagues (2012). Hot spots policing focuses on small geographic areas and concentrations of crime. Hot spots policing per se does not demand detailed analysis of the problem identified and often relies on a law enforcement response. Problem‐oriented policing can focus on small geographic areas (hot spots); however, further analysis is undertaken to determine the creation of the hot spot and responses are tailored to the needs of each hot spot. Further, problem‐oriented policing also examines non‐geographic concentrations of crime – repeat offenders, repeat victims, hot products, and so forth. In short, while problem‐oriented policing at hot spots can be considered a type of problem‐oriented policing, many hot spots policing programs do not use the more systematic methods associated with problem‐oriented policing.

2 The seminal pieces that will be used here are: Braga et al., 1999; Eck and Spelman, 1987; Goldstein, 1979; Goldstein, 1990; Spelman and Eck, 1987; Weisburd et al., 2008, 2010.

3 These journals will include: *Cambridge Journal of Evidence‐Based Policing, Criminology, Criminology and Public Policy, Justice Quarterly, Journal of Research in Crime and Delinquency, Journal of Criminal Justice, Police Quarterly, Policing, Police Practice and Research, British Journal of Criminology, Journal of Quantitative Criminology, Crime and Delinquency, Journal of Criminal Law and Criminology, Policing and Society*.

4 The POP center is included in the GPD's grey literature searches, but given the centrality of the center to the current review topic we opted to double check this source for eligible studies.
