# Supplementary material for: Updated protocol: The effects of problem‐oriented policing on crime and disorder: An updated systematic review
Source: Campbell Syst Rev. 2019 Jul 25;15(1-2):e1005. doi: 10.1002/cl2.1005 (PMC8356314; doi:10.1002/cl2.1005)
Supplement: Supplementary file 1 — Supplementary information [file CL2-15-e1005-s001.docx]

Appendix A: GPD Systematic Search Strategy^[[1]](#footnote-1)^

Search Terms

To ensure optimum sensitivity and specificity, the GPD search strategy utilises a combination of free-text and controlled vocabulary search terms. Because controlled vocabularies and search capabilities vary across databases, the exact combination of search terms and field codes are adapted to each database. Final search syntax for each location will be reported in the final review.

The free-text search terms for the GPD are provided in Table 1 and are grouped by substantive (i.e., some form of policing) and evaluation terminology. Although the search strategy may vary slightly across search locations, it follows a number of general rules:

- Search terms are combined into search strings using Boolean operators “AND” and “OR”. Specifically, terms within each category are combined with “OR”, and categories will be combined with “AND”. For example: (police OR policing OR “law#enforcement”) AND (analy* OR ANCOVA OR ANOVA OR …).
- Compound terms (e.g., law enforcement) are considered single terms in search strings by using quotation marks (i.e., “law*enforcement”) to ensure that the database searches for the entire term rather than separate words.
- Wild cards and truncation codes are used for search terms with multiple iterations from a stem word (e.g., evaluation, evaluate) or spelling variations (e.g., evaluat* or randomi#e).
- If a database has a controlled vocabulary term that is equivalent to “POLICE”, the term is combined in a search string that includes both the policing and evaluation free-text search terms. This approach ensures that the search retrieves documents that do not use policing terms in the title/abstract but have been indexed as being related to policing in the database. An example of this approach is the following search string: (((SU: “POLICE”) OR (TI,AB,KW: police OR policing OR “law*enforcement”)) AND (TI,AB,KW: intervention* OR evaluat* OR compar* OR …)).
- For search locations with limited search functionality, a broad search that uses only the policing free-text terms is implemented.
- Multidisciplinary database searches are limited to relevant disciplines (e.g., include social sciences but exclude physical sciences).
- Search results are refined to exclude specific types of documents that are not suitable for systematic reviews (e.g., newspapers, front/back matter, book reviews).

Table 1. Free-text search terms for the GPD systematic search

| **Policing Search Terms** | **Evaluation Search Terms** | | | |
| --- | --- | --- | --- | --- |
| police  policing  “law*enforcement”  constab*  detective*  sheriff* | analy*  ANCOVA  ANOVA  “ABAB design”  “AB design”  baseline  causa*  “chi#square”  coefficient*  “comparison condition*”  “comparison group*”  “control condition*”  “control group*”  correlat*  covariat*  “cross#section*” | data  effect*  efficacy  eval*  experiment*  hypothes*  impact*  intervent*  interview*  longitudinal  MANCOVA  MANOVA  “matched group”  measure*  “meta-analy*”  “odds#ratio* | outcome*  paramet*  “post-test”  posttest  “post test”  predict*  “pre-test”  pretest  program*  “propensity score*”  quantitative  “quasi#experiment*”  questionnaire*  random*  RCT  regress* | result*  “risk#ratio*”  sampl*  “standard deviation*”  statistic*  studies  study  survey*  “systematic review*”  “t#test*”  “time#series”  treatment*  variable*  variance |

Search Locations

To reduce publication and discipline bias, the GPD search strategy adopts an international scope and involves searching for literature across a number of disciplines (e.g., criminology, law, political science, public health, sociology, social science and social work). The search captures a comprehensive range of published (i.e., journal articles, book chapters, books) and unpublished literature (e.g., working papers, governmental reports, technical reports, conference proceedings, dissertations) by implementing a search strategy across bibliographic/academic, grey literature, and dissertation databases or repositories.

It is noted that there is substantial overlap of the content coverage between many of the databases. Therefore, the *Optimal Searching of Indexing Databases* (OSID) computer program (Neville & Higginson, 2014) has been used to analyse the content crossover for all databases that have accessible content coverage lists. OSID analyses the content coverage and creates a search location solution that provides the most comprehensive coverage via the least number of databases. Another advantage of using OSID when designing a search strategy is the reduction in the number of duplicates that would need to be removed prior to the screening phase. Databases with >10 unique titles are searched in full, whereas databases with ≤10 unique titles were searched only the unique titles and any non-serial content (e.g., reports, conference proceedings). Where a modified search of a database would be more labour-intensive than a full search and export results, a full search of the database is conducted. The final search locations and solutions are reported in Table 2.

Table 2. GPD search locations and protocol (January 1^st^ 1950 – December 2018)

| **INDEXED & ACADEMIC DATABASES** |  | **CONTENT COVERAGE FED INTO OSID?** | **FULL OR MODIFIED SEARCH?** | **SEARCH MODIFICATIONS** |
| --- | --- | --- | --- | --- |
| **ProQuest** | Criminal Justice | Yes | Full | None. |
|  | Dissertation and Theses Database Global | Not Available | Modified | Social Sciences subset. |
|  | Political Science | Yes | Full | None. |
|  | Periodical Archive Online | Yes | Full | None. |
|  | Research Library | Yes | Modified | Social Sciences subset. |
|  | Social Science Journals | Yes | Full | None. |
|  | Sociology | Yes | Modified | Search 2 unique journal titles and non-serial content only. |
|  | Applied Social Sciences Index and Abstracts | Yes | Full | None. |
|  | International Bibliography of the Social Sciences | Yes | Full | None. |
|  | Public Affairs Information Service | Yes | Full | None. |
|  | Social Services Abstracts | Yes | Modified | Search 5 unique journal titles and non-serial content only. |
|  | Sociological Abstracts | Yes | Full | None. |
|  | Worldwide Political Sciences Abstracts | Yes | Modified | Search 9 unique journal titles and non-serial content only. |
| **EBSCO** | Academic Search Premier | Yes | Full | None. |
|  | Criminal Justice Abstracts | Yes | Full | None. |
|  | EconLit | Yes | Full | None. |
|  | MEDLINE with Full-Text | Yes | Full | None. |
|  | Social Sciences Full-Text | Yes | Full | None. |
| **OVID** | International Political Science Abstracts | Not Available | Full | None. |
|  | PsycARTICLES | Yes | Modified | Search 4 unique journal titles only. |
|  | PsycEXTRA | Not Available | Full | None. |
|  | PsycINFO | Yes | Full | None. |
|  | Social Work Abstracts | Not Available | Full | None. |
| **Web of Science** | Current Contents Connect – Social and Behavioural Sciences Edition | Yes | Modified | Search 1 unique journal title and non-serial content only. |
|  | Book Citation Index (Social Sciences and Humanities) | Not Available | Full | None. |
|  | Conference Proceedings Citation Index (Social Sciences and Humanities) | Not Available | Full | None. |
|  | Social Science Citation Index | Yes | Full | None. |
| **Informit** | Australian Attorney General Information Service | Yes | Full | None. |
|  | Australian Criminology Database (CINCH) | Yes | Full | None. |
|  | Australian Federal Police Database | Yes | Full | None. |
|  | Australian Public Affairs Full-Text | Yes | Full | None. |
|  | DRUG | Yes | Full | None. |
|  | Health & Society Database | Yes | Modified | Search unique journal titles and non-serial content only. |
|  | Humanities and Social Sciences Collection | Yes | Full | None. |
| **Gale-Cengage** | Expanded Academic ASAP | Yes | Full | None. |
| **STANDALONE & OPEN ACCESS DATABASES** | Cambridge Journals Online | Yes | Modified | Search 4 unique journal titles in Law and Political Science collections and full search of Social Studies collection. |
|  | Directory of Open Access Journals | Yes | Full | None. |
|  | HeinOnline | Yes | Modified | Law Journals Online collection only. |
|  | JSTOR | Yes | Modified | Search unique titles across the Law, Political Science, Public Health, Public Policy, Social Work and Sociology collections only. The Criminal Justice collection had no unique content and so will be excluded from the search. Only 10% of content in this database have abstracts and a full-text search returns >250,000 results because of inability to construct complex search strings. Therefore, a modified search of the unique titles across these collections will be more pragmatic than a full search of the database. |
|  | Oxford Scholarship Online | Yes | Full | None. |
|  | Sage Journals Online and Archive (Sage Premier) | Yes | Modified | Search 5 unique journal titles and non-serial content only. |
|  | ScienceDirect | Yes | Full | None. |
|  | SCOPUS | Yes | Full | None. |
|  | SpringerLink | Yes | Full | Although this database has low uniqueness when combined with the full set of databases, a full search using only the policing search terms will be more pragmatic than a modified search on unique titles because of the restricted search functionality of this database. |
|  | Taylor & Francis Online | Yes | Modified | Although this database has low uniqueness when combined with the full set of databases, a full search using only the policing search terms will be more pragmatic than a modified search on unique titles because of the restricted search functionality of this database. |
|  | Wiley Online Library | Yes | Full | None. |
|  | California Commission on Peace Officer Standards & Training Library | No | Full | None. |
|  | Cochrane Library | No | Full | None. |
|  | CrimeSolutions.gov | No | Full | None. |
|  | Database of Abstracts of Reviews of Effectiveness (DARE) | No | Full | None. |
|  | FBI – The Fault (Reports and Publications) | No | Full | None. |
|  | Evidence-Based Policing Matrix | No | Full | None. |
|  | International Initiative for Impact Evaluation Database (3ie) | No | Full | None. |
|  | National Criminal Justice Reference Service | No | Full | None. |
|  | Safety Lit Database | No | Full | None. |
|  | Australian Institute of Criminology | No | Full | None. |
|  | Bureau of Police Research and Development (India) | No | Full | None. |
|  | Canadian Police Research Catalogue | No | Full | None. |
|  | Centre for Problem-Oriented Policing | No | Full | None. |
|  | College of Policing (including POLKA and Crime Reduction Toolkit) | No | Full | None. |
|  | European Police College (CEPOL) | No | Full | None. |
|  | Evidence for Policy and Practice Information and Coordinating Centre | No | Full | None. |
|  | National Research Institute of Police Science (Japanese) | No | Full | None. |
|  | Office of Community Oriented Policing Services | No | Full | None. |
|  | Police Executive Research Forum (US) | No | Full | None. |
|  | Police Foundation (US) | No | Full | None. |
|  | Tasmania Institute of Law Enforcement Studies (Australia) | No | Full | None. |
|  | Policing Online Information System (POLIS, Europe) | No | Full | None. |
|  | Scottish Institute for Policing Research | No | Full | None. |
|  | Centre of Excellence in Policing and Security (Australian, now archived) | No | Full | None. |

Appendix B: GPD Systematic Compilation Strategy

Inclusion Criteria

Each record captured by the GPD systematic search must satisfy all inclusion criteria to be included in the GPD: timeframe, intervention and research design. There are no restrictions applied to the types of outcomes, participants, settings or languages considered eligible for inclusion in the GPD.

Types of interventions

Each document must contain an impact evaluation of a policing intervention. Policing interventions are defined as some kind of a strategy, program, technique, approach, activity, campaign, training, directive, or funding/organisational change that involves police in some way (other agencies or organisations can be involved). Police involvement is broadly defined as:

- Police initiation, development or leadership
- Police are recipients of the intervention or the intervention is related, focused or targeted to police practices
- Delivery or implementation of the intervention by police

Types of study designs

The GPD includes quantitative impact evaluations of policing interventions that utilise randomised experimental (e.g., RCTs) or quasi-experimental evaluation designs with a valid comparison group that does not receive the intervention. The GPD includes designs where the comparison group receives ‘business-as-usual’ policing, no intervention or an alternative intervention (treatment-treatment designs).

The specific list of research designs included in the GPD are as follows:

- Systematic reviews with or without meta-analyses
- Cross-over designs
- Cost-benefit analyses
- Regression discontinuity designs
- Designs using multivariate controls (e.g., multiple regression)
- Matched control group designs with or without pre-intervention baseline measures (propensity or statistically matched)
- Unmatched control group designs with pre-post intervention measures which allow for difference-in-difference analysis
- Unmatched control group designs without pre-intervention measures where the control group has face validity
- Short interrupted time-series designs with control group (less than 25 pre- and 25 post-intervention observations)
- Long interrupted time-series designs with or without a control group (≥25 pre- and post-intervention observations)
- Raw unadjusted correlational designs where the variation in the level of the intervention is compared to the variation in the level of the outcome

The GPD excludes single group designs with pre- and post-intervention measures as these designs are highly subject to bias and threats to internal validity.

Systematic Screening

To establish eligibility, records captured by the GPD search progress through a series of systematic stages which are summarised in Figure 1, with additional detail provided in the following subsections.

All research staff working on the GPD undergo standardised training before beginning work within any of the stages detailed below. Staff then complete short training simulations to enable an assessment of their understanding of the GPD protocols and highlight any areas for additional training. In addition, random samples of each staff’s work are regularly cross-checked to ensure adherence to protocols. Disagreements about screening decisions between staff are mediated by either the project manager or GPD chief investigators.

Title and abstract screening

After removing duplicates, the title and abstract of records captured by the GPD systematic search is screened by trained research staff to identify potentially eligible research that satisfies the following criteria:

- Document is dated between 1950 – present
- Document is unique (i.e., not a duplicate)
- Document is about police or policing
- Document is an eligible document type (e.g., not a book review)

Records are excluded if the answer to any one of the criteria is unambiguously ‘No’, and will be classified as potentially eligible otherwise. Records classified as eligible at the title and abstract screening stage progress to full-text document retrieval and screening stages.

Full-text eligibility screening

Wherever possible, a full-text electronic version of an eligible record is imported into *SysReview* (review management software; Higginson & Neville, 2015). For records without an electronic version, a hardcopy of the record is located to enable full-text eligibility screening. The full-text of each document is screened to identify studies that satisfy the following criteria:

- Document is dated between 1950 – present
- Document is unique
- Document reports a quantitative statistical comparison
- Document reports on policing evaluation
- Document reports in a quantitative impact evaluation of a policing intervention
- Evaluation uses an eligible research design

*Figure 1.* GPD systematic compilation process

**Appendix C: POP META ANALYSIS CODING SHEET**

**Reference Information**

1. Document ID: __ __ __ __

2. Study author(s): ____________________

3. Study title: _______________________

4a. Publication type: ______

1. Book

2. Book chapter

3. Journal article (peer reviewed)

4. Thesis or doctoral dissertation

5. Government report (state/local)

6. Government report (federal)

7. Police department report

8. Technical report

9. Conference paper

10. Other (specify)

4b. Specify (Other)_____________________

5. Publication date (year): ______________

6a. Journal Name: ____________________

6b. Journal Volume: _______________

6c. Journal Issue: ____________

7. Date range of research (when research was conducted):

Start: ____________

Finish: ____________

8. Source of funding for study: ___________________

9. Country of publication: ___________________

10. Date coded: ___________

11. Coder’s Initials: __ __ __

**Describing the Problem(s)**

12. How did the problem(s) come to the attention of the police? (Select all that apply)

1. Crime analysis unit

2. Citizen meeting/organization

3. Officer observation/suggestion

4. Other government agency

5. Funding agency

6. Researcher

7. Other (specify)

12b. Specify (Other) _____________

13. What was the environment where the problem(s) occurred? (Select all that apply)

1. Residential

2. Recreational (bars, restaurants, parks)

3. Offices

4. Retail

5. Industrial

6. Agricultural

7. Education

8. Human service (jails, courts, hospitals)

9. Public ways

10. Transport (buses, airports)

11. Open/transitional (construction sites, abandoned buildings)

12. Citywide/no particular environment specified

14a. What type of event(s) make up the problem(s)? ______

1. Predatory crimes against persons (sexual assault, robbery, homicide)

2. Predatory crimes against property (vandalism, auto theft)

3. Illegal service crimes (prostitution, selling drugs)

4. Public disorder crimes (disorderly conduct, drunkenness)

5. Vehicular/traffic offenses

6. Status crimes

7. Hard drug use

8. Overall crime/disorder

9. Other (specify)

14b. Specify (Other) ___________

15. Specifically, what event(s) makes up the problem(s)? ______________________________________________________________________________

____________________________________________________________________________________________________________________________________________________________

16. The events making up the problem(s) primarily center on which part of the problem analysis/crime triangle?

1. Offenders

2. Victims/targets

3. Guardians or managers

4. Places/geographic areas

17a. What data sources were used for analysis of the selected problem? (Select all that apply)

1. Official crime data

2. Arrest information

3. Surveys of people (non-offenders)

4. Surveys of places or environments

5. Interviews and discussions with people (non-offenders)

6. Interviews of offenders

7. Literature examination

8. Consultation with government agencies

9. Consultations with businesses

10. Consultations with community organizations

11. Other (specify)

17b. Specify (Other)___________________

18. What was the level/intensity of problem analysis?

1. No analysis

2. Shallow or cursory analysis (looked at official data)

3. Moderate analysis (looked at official data with analysis by time of day, day of week

etc.)

4. In-depth analysis (3 above, as well as other problem analysis with other data)

5. Authors do not provide sufficient detail to make an assessment

**Describing the Response**

19. At what unit of analysis was the treatment delivered/intervention primarily directed at?

1. Micro place (e.g., hot spot)

2. Meso area (e.g., neighborhoods)

3. Large area (e.g., entire city)

4. Individual offender

5. Individual victim

6. Group of offenders (e.g., gang)

7. Group of victims

8. Individual guardian or manager

9. Group of guardians or managers

10. Entire population (no types of individuals or groups specified)

11. Other (specify)

20a. Did the evaluation use the same unit of analysis as the unit the intervention was directed at?

1. Yes

2. No

20b. If No, specify the unit of analysis for the evaluation

1. Micro place (e.g., hot spot)

2. Meso area (e.g., neighborhoods)

3. Large area (e.g., entire city)

4. Individual offender

5. Individual victim

6. Group of offenders (e.g., gang)

7. Group of victims

8. Individual guardian

9. Group of guardians

10. Entire population (no types of individuals or groups specified)

11. Other (specify)

21. Briefly describe the response(s) implemented

____________________________________________________________________________________________________________________________________________________________

22a. What techniques of situational crime prevention were used in the implementation of the response? (Select all that apply)

1. Increasing the effort of crime

2. Increasing the risks of crime

3. Reducing the rewards of crime

4. Reducing provocations

5. Removing excuses for crime

6. Situational crime prevention used, but specific techniques not specified

7. N/A- Situational crime prevention not used

8. Other

22b. Specify (Other)___________________

23a. What groups (other than the police) were involved in the implementation of the response? (Select all that apply)

1. Neighborhood associations/organizations

2. Government organizations/agencies

3. Social service agencies

4. Commercial establishments/businesses

5. National organizations with an interest in the problem (e.g. MADD)

6. Individual residents

7. Other police agencies

8. Other criminal justice agencies

9. Other (specify)

23b. Specify (Other)___________________

24a. At what level of the police department was the response implemented? _____

1. Entire department/all officers involved

2. Certain precincts/districts involved

3. Special unit (i.e. community policing unit) involved

4. Select few officers in specific area involved

5. Other (specify)

6. N/A (not mentioned)

24b. Specify (Other)___________________

***Implementation of Response***

25. What did the evaluation indicate about the implementation of the response? _____

1. There were no reported implementation issues

2. There were minor implementation issues

3. There were more substantial implementation issues

4. There were major implementation issues/the project was not implemented as planned

5. Unclear/no process evaluation included

26. If the process evaluation indicated there were problems with implementation of the response, describe these problems:

__________________________________________________________________________________________________________________________________________________________________________________________________________________________________________

***Location of the intervention***

27. Country where study was conducted: __________________

28. City (and state/province, if applicable) where study was conducted: _________________

*The following questions refer to the area receiving treatment:*

29a. Geographic area receiving treatment: ______

1. Micro place (street segments/blocks)

2. Neighborhood/police beat

3. Police district/precinct

4. Entire city

5. Other (specify)

29b. Specify (Other)___________________

30. What is the exact geographic area receiving treatment? ______________________________________________________________________________

*The following refer to the area not receiving treatment*

31a. Geographic area NOT receiving treatment: ______

1. Micro place (street segments/blocks)

2. Neighborhood/police beat

3. Police district/precinct

4. Entire city

5. Other (specify)

31b. Specify (Other)___________________

32. What is the exact geographic area not receiving treatment? ____________________________________________________________________________

**Methodology/Research design:**

33a. Type of study: _____

1. Randomized experiment

2. Nonequivalent control group (quasi-experimental)

3. Multiple time series (quasi-experimental)

4. Interrupted time series

5. Other (specify)

33b. Specify (Other)___________________

33c. If a quasi-experiment, how was matching of groups achieved?

1. Propensity score matching
2. Identification of matching areas or persons through regression analyses
3. Statistical tests of mean differences among demographic and other relevant variables
4. Comparison of descriptive statistics with no statistical test of differences across groups
5. Comparison to the rest of a jurisdiction or population that did not receive the treatment

33d. Specify (Other)___________________

34a. Were any sources of nonequivalence or bias reported or implied in the application of the intervention or its analysis (i.e. threats to internal validity)?

1. Yes

2. No

34b. If yes, what sources of nonequivalence or bias were identified? (check all that apply and explain)

1. Extraneous events or factors occurring during the intervention period; historical artifacts

2. Selection of treatment area based on high baseline crime rate

3. Measurement confounds (measure changes over time)

4. Differential attrition, breakdown of randomization, or contamination of control group

5. Pre-test analyses indicated nonequivalence between treatment and control groups

6. Statistical analyses failed to adjust for nonequivalence at baseline

7. Inappropriate statistical analysis for design

8. Any outcomes measured by reporters that did not have corresponding outcome measures in the results

9. Other threats to internal validity (specify)

34c. Explain any yes responses checked in 34b.

______________________________________________________________________________________________________________________________________________________________________________________________________

35. Did the researcher assess the quality of the data collected?

1. Yes

2. No

36a. Did the researcher(s) express any concerns over the quality of the data?

1. Yes

2. No

36b. If yes, explain ____________________________________________________________________________________________________________________________________________________________

37a. Does the evaluation data correspond to the initially stated problem? (i.e. if the problem is fear of crime, does the evaluation data look at whether fear of crime decreased)

1. Yes

2. No

37b. If no, explain the discrepancy: ____________________________________________________________________________________________________________________________________________________________

***Outcomes reported*** *(Note that for each outcome, a separate coding sheet is required)*

38. How many crime/disorder outcomes are reported in the study? _____

39. What is the specific outcome recorded on this coding sheet?

_______________________________________________________________

40. Was it the primary outcome of the study? _______

1. Yes

2. No

3. Can’t tell/researcher did not prioritize outcomes

***Dependent Variable***

41a. What type of data was used to measure the outcome covered on this coding sheet? ____

1. Official data (from the police)

2. Researcher observations

3. Self-report surveys

4. Other (specify)

41b. Specify (Other)___________________

42a. If official data was used, what specific type(s) of data were used? (Select all that apply)

1. Calls for service (911 calls)/crime reports

2. Arrests

3. Incident reports

4. Level of citizen complaints

5. Other (specify)

6. N/A (official data not used)

42b. Specify (Other)___________________

43a. If researcher observations were used, what types of observations were taken? (Select all that apply)

1. Physical observations (e.g. observed urban blight, such as trash, graffiti)

2. Social observations (e.g. observed disorder, such as loitering, public drinking)

3. Other observations (specify)

4. N/A (researcher observations not used)

43b. Specify (Other)___________________

44a. If self-report surveys were used, who was surveyed? (Select all that apply)

1. Residents/community members

2. Business owners

3. Elected officials

4. Government/social service agencies

5. Other (specify)

6. N/A (self-report surveys not used)

44b. Specify (Other)___________________

**Effect size/Reports of statistical significance**

***Sample size***

45. Based on the unit of analysis for this outcome, what is the total sample size in the analysis? ________

46. What is the total sample size of the treatment group (group that receives the response)? _______

47. What is the total sample size of the control group (if applicable)? _____

48a. Was attrition a problem in the analysis for this outcome?

1. Yes

2. No

48b. If attrition was a problem, provide details (e. g. how many cases lost and why they were lost).

__________________________________________________________________________________________________________________________________________________________________________________________________________________________________________

49a. What do the sample sizes above refer to?

1. Crimes

2. People

3. Geographic areas

4. Other (specify)

49b. Specify (other) ________________

***Effect Size Data***

50. Raw difference favors (i.e. shows more success for):

1. Treatment group

2. Control group

3. Neither (exactly equal)

9. Cannot tell (or statistically insignificant report only)

51. Did a test of statistical significance indicate statistically significant differences between either the control and treatment groups or the pre and post tested treatment group? ____

1. Yes

2. No

3. Can’t tell

4. N/A (no testing completed)

52. Was a standardized effect size reported?

1. Yes

2. No

53. If yes, what was the effect size? ______

54. If yes, page number where effect size data is found ________

55. If no, is there data available to calculate an effect size?

1. Yes

2. No

56a. Type of data effect size can be calculated from:

1. Means and standard deviations

2. *t*-value or *F*-value

3. Chi-square (df=1)

4. Frequencies or proportions (dichotomous)

5. Frequencies or proportions (polychotomous)

6. Other (specify)

56b. Specify (other) _________

*Pre-post Study Counts*

57a. Pre-period number of events for current outcome in target area _______

57b. During intervention-period number of events for current outcome in target area ______

57c. Post-period number of events for current outcome in target area ______

57d. Pre-period number of events for current outcome in comparison area _______

57e. During intervention-period number of events for current outcome in comparison area _____

57f. Post-period number of events for current outcome in comparison area ______

57g. Did the evaluation control for validity by using multivariate methods (i.e. regression) to assess the impact of the program?

1. Yes

2. No

57h. If yes, did this analysis find that the intervention reduced the outcome at a statistically significant level?

1. Yes

2. No

3. N/A

*Means and Standard Deviations*

58a. Treatment group mean. _____

58b. Control group mean. _____

59a. Treatment group standard deviation. _____

59b. Control group standard deviation. _____

*Proportions or frequencies*

60a. *n* of treatment group with a successful outcome. _____

60b. *n* of control group with a successful outcome. _____

61a. Proportion of treatment group with a successful outcome. _____

61b. Proportion of treatment group with a successful outcome. _____

*Significance Tests*

62a. *t*-value _____

62b. *F*-value _____

62c. Chi-square value (*df*=1) _____

*Calculated Effect Size*

63a. Effect size ______

63b. Standard error of effect size _____

**Conclusions made by the author(s)**

*Note that the following questions refer to conclusions about the effectiveness of the intervention in regards to the current outcome/problem being addressed on this coding sheet.*

64. Conclusion about the impact of the intervention? _____

1. The authors conclude intervention associated with a crime decline

2. The authors conclude intervention not associated with a crime decline

3. Unclear/no conclusion stated by authors

65. Did the assessment find evidence of a geographic displacement of crime? ______

1. Yes

2. No

3. Not tested

66a. Did the assessment find evidence of other non-geographic types of displacement of crime? _____

1. Yes

2. No

3. Not tested

66b. If yes, specify what types of displacement were found

______________________________________________________________________________________________________________________________________________________

67. Additional notes about conclusions:

____________________________________________________________________________________________________________________________________________________________

68. Additional notes about study:

____________________________________________________________________________________________________________________________________________________________

**Appendix D: List of Policing Experts to be Consulted**

List of policing scholars and practitioners contacted to identify any studies we missed (Note: Job titles reflect employer as of January 2019)

| ***Name*** | ***Employer*** |
| --- | --- |
| Bayley, David | University at Albany, State University of New York |
| Boba Santos, Rachel | Radford University |
| Bobo, Lawrence | Harvard University |
| Braga, Anthony | Northeastern University |
| Bynum, Tim | Michigan State University |
| Capowich, George | Loyola University, New Orleans |
| Clarke, Ronald | Rutgers-Newark, The State University of New Jersey |
| Cordner, Gary | Kutztown University of Pennsylvania |
| Davis, Rob | The National Police Foundation |
| Forst, Brian | American University |
| Glensor, Ron | Arizona State University |
| Goldstein, Herman | University of Wisconsin Law School |
| Greene, Jack | Northeastern University |
| Groff, Elizabeth | Temple University |
| Hope, Tim | University of Salford |
| Kelling, George | Manhattan Institute (x) |
| Kennedy, David | John Jay College of Criminal Justice |
| Klinger, David A. | University of Missouri- St. Louis |
| Knutsson, Johannes | Norwegian Police University College |
| Koper, Chris | George Mason University |
| Lauritsen, Janet | University of Missouri- St. Louis |
| Laycock, Gloria | Jill Dando Institute, University College London |
| Lum, Cynthia | George Mason University |
| Maclin, Tracey | Boston University Law School |
| Maguire, Ed | Arizona State University |
| Manning, Peter | Northeastern University |
| Mastrofski, Stephen | George Mason University |
| Mazerolle, Lorraine | University of Queensland, Australia |
| McGarrell, Ed | Michigan State University |
| Meares, Tracey | Yale University Law School |
| Mills, Andy | Santa Cruz Police Department |
| Moore, Mark | Harvard University |
| Newman, Graeme | University at Albany, State University of New York |
| Peterson, Ruth | Ohio State University |
| Ratcliffe, Jerry | Temple University |
| Ready, Justin | Griffiths University |
| Roehl, Janice | Justice Research Center |
| Rosenbaum, Dennis | University of Illinois at Chicago |
| Sampson, Rana | Union Bank, San Diego |
| Saville, Gregory | AlterNation Consulting |
| Schmerler, Karin | San Diego County District Attorney’s Office |
| Schultze, Phyllis | Rutgers-Newark, The State University of New Jersey |
| Scott, Michael | Arizona State University |
| Sharp, Elaine B. | University of Kansas |
| Sherman, Lawrence | University of Maryland |
| Silverman, Eli | John Jay College of Criminal Justice |
| Skogan, Wesley | Northwestern University |
| Skolnick, Jerome | New York University Law School |
| Sousa, William | University of Nevada, Las Vegas |
| Spelman, William | University of Texas |
| Stephens, Darrel | Darrel Stephens Group, LLC |
| Stephenson, Paul | Embrace Child Victims of Crime |
| Tilley, Nick | Nottingham Trent University |
| Tita, George | University of California, Irvine |
| Travis, Jeremy | Laura and John Arnold Foundation |
| Uchida, Craig | Justice and Security Strategies |
| Walker, Samuel | University of Nebraska, Omaha |
| Weisel, Deborah Lamm | North Carolina State University |
| Wellford, Charles | University of Maryland |
| Welsh, Brandon | Northeastern University |
| Willis, James | George Mason University |
| Worden, Robert | University at Albany, State University of New York |

1. Appendices A and B are taken directly from Higginson, A., Eggins, E., Mazerolle, L. and Stanko, E. (2015). *The Global Policing Database [Database and Protocol].* [↑](#footnote-ref-1)
